# Laser resection of endobronchial hamartoma via fiberoptic bronchoscopy

**DOI:** 10.4103/0970-2113.68329

**Published:** 2010

**Authors:** Satya Prakash Rai, Ashok Police Patil, Puneet Saxena, Amulyajit Kaur

**Affiliations:** *Department of Respiratory Medicine, Military Hospital (Cardio-Thoracic Centre), Pune, India*; 1 *Department of Pathology, Military Hospital (Cardio-Thoracic Centre), Pune, India*

**Keywords:** Diode laser, endobronchial hamartoma, fiberoptic bronchoscope, laser resection

## Abstract

Endobronchial hamartoma is a rare benign tumor of lung that may present with symptoms of airway obstruction with wheezing, stridor, recurrent pneumonia or atelectasis. We report a case of a patient with endobronchial hamartoma, recurrent pneumonia, who presented to us with sputum smear and culture positive pulmonary tuberculosis. He was treated with antitubercular treatment and endobronchial hamartoma was resected completely by diode laser through fiberoptic bronchoscope.

## INTRODUCTION

Pulmonary hamartomas are rare benign tumors of the lung with an incidence of 0.25% to 0.32%.[1] However, they are rare within the central airways. In the largest published series of pulmonary hamartomas, Gjevre *et al*.[2] analyzed 215 cases of hamartoma, of which only 1.4% were located endobronchially. The authors concluded that the hamartoma was a benign neoplasm and frequently asymptomatic. Endobronchial hamartoma is a special form of the intra pulmonary hamartoma, which originates from a large bronchus, grows into the lumen and causes bronchial obstruction. They are of mesenchymal tissue origin.

Endobronchial hamartomas have been successfully removed both endoscopically and surgically without significant complication.[3–4] Endobronchial laser resection has an important role, especially in patients who refuse surgery or who are not surgical candidates. Only lasers with wavelengths that can be delivered through an optical fiber are suitable for laser bronchoscopy. Neodymium-yttrium aluminum garnet (Nd: YAG) and diode laser both have been used for laser photoresection. Diode laser is much smaller than traditional YAG laser and are solid state devices hence compact and portable.[5] Both rigid and flexible bronchoscope can be used for laser resection, the key deciding factor being technical experience of the operator.

## CASE REPORT

A 40-year-old male presented to us with two weeks history of cough, low grade fever, anorexia and three kg weight loss. Contrast enhanced computerized tomography (CECT) chest and bronchoscopy had shown large endobronchial mass occluding left main bronchus in Jun 2006 during evaluation for obstructive pneumonia (left). Endobronchial biopsies done twice were inconclusive. Since then he had received antibiotics for recurrent pneumonia (once every two to three months). On examination he was found to have features of collapse consolidation left lung with monophonic wheeze over left hemithorax. Other systems were normal.

**Figure 1:**
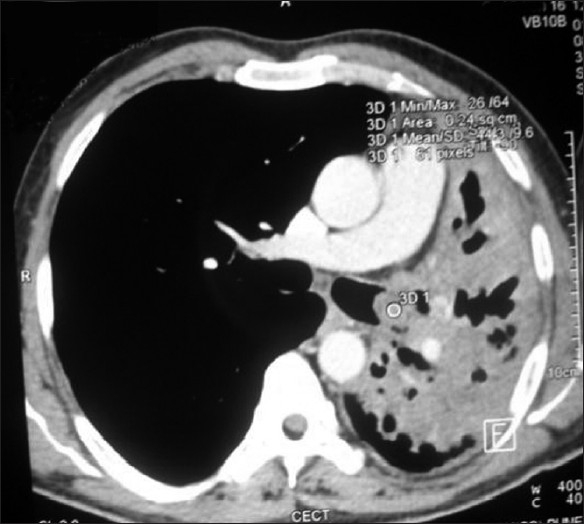
CT scan of chest showing endobronchial mass obstructing left main bronchus and collapse consolidation of left lung

**Figure 2:**
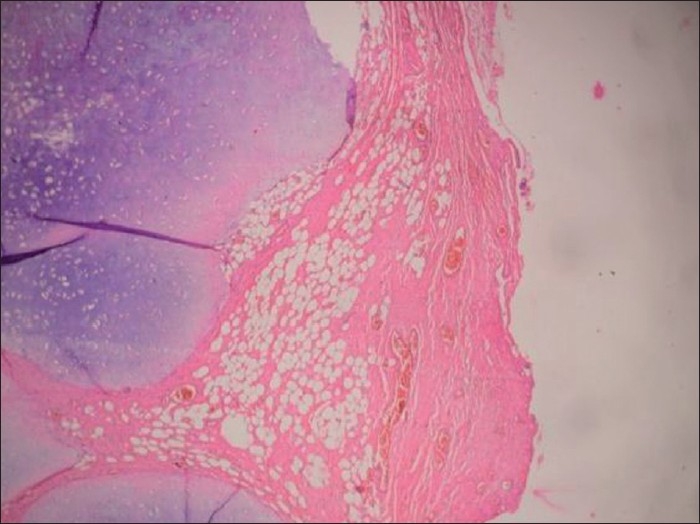
Histopathology of bronchial biopsy showing variably sized disorganized lobules of hyaline cartilage with an admixture of mature benign adipose tissue and a few fine blood vessels (H and E, ×200)

**Figure 3:**
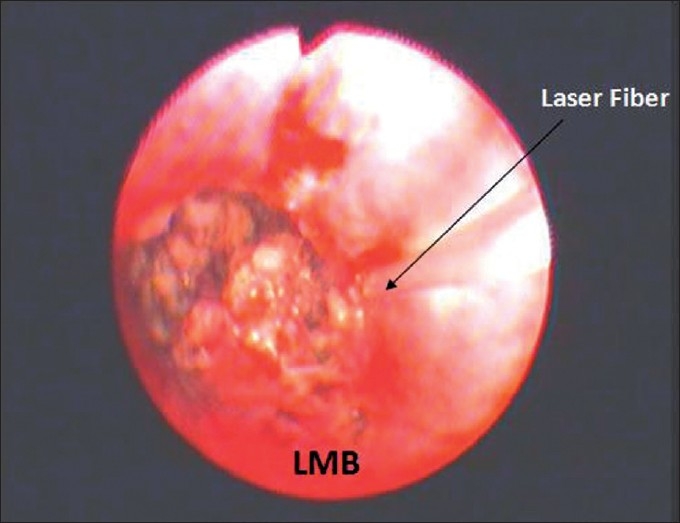
Bronchoscopic appearance of the endobronchial mass being resected with diode laser

**Figure 4:**
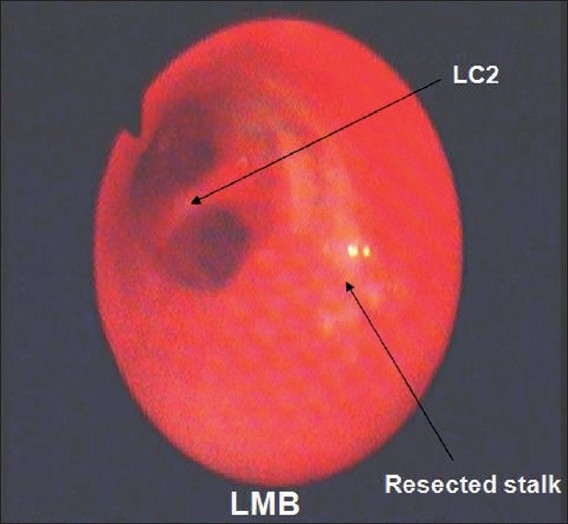
Bronchoscopic appearance of the left main bronchus after laser procedure showing patent segmental bronchi and stalk of the resected mass

The chest radiograph showed collapse of left lower lobe with fibrocavitary lesion involving left upper lobe. Sputum smear and MTB culture were positive and it was sensitive to primary antitubercular drugs. Routine hematological, urinalysis and biochemical parameters were normal. CECT chest revealed endobronchial mass obstructing left main bronchus and collapse consolidation of left lung [Figure 1]. Fiberoptic bronchoscopy showed a pink colored smooth, fleshy, pedunculated mass in the left main bronchus about 2 cm from the carina, almost completely obstructing the bronchus. Bronchial biopsy on microscopy showed disorganized lobules of cartilage and adipose tissue confirming the diagnosis of chondroid hamartoma [Figure 2]. He was treated with antitubercular treatment (3EHRZ/3HR) and the endobronchial mass was resected endoscopically by diode laser. SST Medysys Diode laser was used for resection through flexible fiberoptic bronchoscope, which was done in endoscopy room under conscious sedation and topical anesthesia. The laser fiber was passed through suction channel of flexible bronchoscope and laser was applied in contact fashion at 6 watt power setting in 0.5 seconds pulses [Figure 3]. Mechanical debulking of tumor was done using forceps and dormia basket. The debris, clot and secretions were removed by suction and biopsy forceps. We were able to achieve complete removal of mass in two sittings (total duration 4 hours). There was no complication. Repeat bronchoscopy done after four weeks showed completely patent normal airway [Figure 4].

## DISCUSSION

Hamartomas are lesions which represent abnormal mixtures of tissue elements or an abnormal proportion of a single element normally present in an organ resulting in changes that are tumor like but not neoplastic; however, chondroid hamartomas despite their popular name, are more likely to be benign connective tissue neoplasms rather than tumor like malformations.[6] Pulmonary hamartomas are rare benign tumors of lung and endobronchial hamartomas are even rarer. Most hamartomas are discovered during adulthood with a peak incidence in the sixth to seventh decade.[7] Their rarity in childhood and continued growth in adulthood and the identification of chromosomal rearrangements (6p21 and 12q14 -15) similar to those found in lipomas and leiomyomas favors a neoplastic nature.[8] There is a 2 to 3:1 male prevalence.[2] Majority of the endobronchial hamartomas are isolated lesions, but they do arise in combination with parenchymal hamartomas. They are benign lesions with very low risk of malignancy and a low rate of recurrence. Clinically most parenchymal hamartomas are asymptomatic.[2–4] Endobronchial hamartomas present usually with symptoms of airway obstruction with wheezing and stridor. Many cases have been misdiagnosed as asthma until the lesions were identified at bronchoscopy or at CT chest. They may present with recurrent pneumonia or atelectasis secondary to endobronchial obstruction as seen in our patient.

Bronchoscopically, the lesions are smooth, fleshy, pedunculated mass that may be tan to pink. The lesions are often polypoid, either sessile or with a thin pedicle. Radiographic findings[9–10] include soft tissue masses within the central airways. Secondary signs include lung hyperinflation, recurrent pneumonia, collapse and bronchiectasis due to airway obstruction. On CT scan these lesions are described as rounded soft tissue masses that frequently exhibit calcification and fat density for adipose tissue which was not evident in our patient.

Histologically the mesenchymal components of the endobronchial hamartomas are highly varied. There is predominance of adipose tissue over other mesenchymal components. They have also been found to contain cartilage, myxomatous connective tissue, smooth muscle and epithelial structures.[6] The main differential, in our patient, is of a chondroma because of predominant cartilage tissue. A chondroma is entirely composed of cartilage tissue; this lesion, however, had an admixture of benign mesenchymal tissues, i.e. hyaline cartilage and adipose tissue.

The mainstay of treatment for endobronchial hamartoma is surgical resection with particular focus on lung sparing or bronchoplastic surgery. Endoscopic resection of mass by laser has been described earlier.[3–4,7] It can be performed with a rigid or flexible bronchoscope. If the lung has been collapsed for more than four to six weeks, chances of expansion of lung after removal of obstruction are less. Patients who developed obstructive pneumonia leading to fibrosis, cicatrization collapse and bronchiectesis of long duration (as seen in our patient) are less likely to benefit. They are candidates for resection surgery. As our patient was unwilling for surgery and duration of obstructive pneumonia was not known, we successfully carried out laser resection of endobronchial mass as a palliative measure, in addition to anti-tubercular treatment.
